# Genomic landscape of pediatric germ cell tumors reveals oncogenic mutations and copy number alterations

**DOI:** 10.3389/fonc.2026.1689022

**Published:** 2026-02-16

**Authors:** Janaina Mello Soares Galvão, Ana Flavia Souza Peres Bezerra, Felipe Antonio de Oliveira Garcia, Ana Glenda Santarosa Vieira, Eduardo Caetano Albino da Silva, André van Helvoort Lengert, Rui Manuel Reis, Luiz Fernando Lopes, Adriane Feijó Evangelista, Mariana Tomazini Pinto

**Affiliations:** 1 Molecular Oncology Research Center, Barretos Cancer Hospital, São Paulo, Brazil; 2 Department of Pathology, Barretos Cancer Hospital, São Paulo, Brazil; 3 Life and Health Sciences Research Institute (ICVS) Medical School, University of Minho, Braga, Portugal; 4 Children’s Cancer Hospital, Barretos Cancer Hospital, São Paulo, Brazil; 5 Saint Jude Global, Saint Jude Research Center, Memphis, TN, United States

**Keywords:** germ cell tumor, pediatric tumor, whole-exome sequencing, mutation, copy number alteration

## Abstract

**Introduction:**

Germ cell tumors (GCTs) are rare neoplasms affecting approximately 3.5% of all pediatric patients, with diverse histological subtypes. Despite their clinical and biological heterogeneity, pediatric GCTs generally exhibit a low mutational burden. Compared to adult GCTs, however, the molecular characterization of pediatric cases remains limited, hindering the development of targeted therapeutic strategies. Therefore, we aimed to elucidate the genomic landscape of pediatric GCT patients via whole exome sequencing (WES).

**Methods:**

WES was performed in 16 pediatric GCTs and respective matched normal samples, including ten ovarian, five testicular, and one mediastinal tumor. The somatic alterations found were described and compared with the clinicopathological characteristics, as well as related to molecular databases.

**Results:**

The somatic mutations found resemble those observed in adult GCTs and recent pediatric GCTs studies. Genes with predicted oncogenic variants were found in seven samples (43.75%) out of 16 and included *KIT* (12.5%), *KRAS* (6.25%), *MTOR* (12.5%), *PIK3CA* (6.25%), *AKT2* (6.25%), *LARP4B* (6.25%), and *ACSL6* (6.25%). Copy number alterations were identified on chromosomes 4, 7, 8, 10, 12, 21, and 22, with amplification of *CDKN1B*, *KRAS*, *CCND2*, *ETV6*, and *KDM5A* genes, and deletions of *KIT* and *PTEN* genes. Clinically significant mutations (*KIT*: Asp816Val, Ala829Pro; and *KRAS*: Gln61Leu) suggest potential therapeutic targets for GCT, while *MTOR*, *PIK3CA*, and *AKT2* emerge as candidates for targeted therapy.

**Discussion:**

These findings provide insights into the genomic alterations of pediatric GCTs and emphasize the potential for targeted therapies.

## 1 Introduction

Germ Cell Tumors (GCTs) are a rare type of cancer, accounting for less than 1% of tumors diagnosed in adults (1) and 3.5% in children (2). Most of these tumors can be cured with initial cisplatin-based chemotherapy, but 20% to 30% of patients demonstrate cisplatin resistance (3), and approximately 15% will relapse (4).

GCTs are a heterogeneous series of tumors comprising seven histologic subtypes subdivided into two major group: 1) Tumors which resemble undifferentiated primordial germ cells: teratomas (which can be benign or malignant depending on its cell’s maturity), seminomas, dysgerminomas, and germinomas; 2) Tumors which show cellular differentiation: yolk sac tumor, embryonal carcinoma, and choriocarcinoma (5). Each subtype requires specific management, and learning about their mutational features will provide novel therapeutic targets.

Compared to other solid tumors, GCTs show a relatively silent mutational landscape (6). Moreover, recent analysis revealed that pediatric cancers harbor different genetic events than adult tumors, presenting a considerably lower tumor mutation burden opposed to common adult cancers (7, 8).

The molecular characteristics of GCT are believed to differ between adult and pediatric patients. However, some studies with GCT patients have identified driver alterations in three genes involved in cell differentiation and proliferation, *KIT*, *KRAS*, and *NRAS* (3, 9, 10) and the *TP53* gene correlated with the overall survival in GCT patients, although previous studies showed that most patients had the wild-type *TP53* (11). Some of these genes, such as *KIT* and *KRAS*, are being studied as therapeutic targets in other tumor types and have ongoing trials, underscoring their potential relevance in precision oncology (12–15).

Understanding the mutational landscape of GCTs offers an opportunity to explore targeted therapies, which could improve outcomes for patients who exhibit cisplatin resistance or relapse. With the increasing role of precision medicine in oncology, identifying actionable genetic alterations in pediatric GCTs may pave the way for more individualized and effective treatment strategies. In this sense, this study aimed to evaluate the most frequent alterations in the coding regions of genes (exome) and assess their pathogenicity in pediatric patients diagnosed with GCTs.

## 2 Materials and methods

### 2.1 Study population

This retrospective study included 16 pediatric patients diagnosed with GCTs and treated at Barretos Cancer Hospital (BCH). It included 10 ovarian, five testicular, and one mediastinum. The pair tumor and blood samples were obtained from biopsy or surgery at diagnosis and were immediately processed and stored at − 80 °C in the Barretos Cancer Hospital Biobank (16).

This study was approved by the Barretos Cancer Hospital’s local ethics committee (IRB/Project No. 1405/2017). Due to the considerable challenges in obtaining consent from participants - especially as some individuals in this cohort are deceased - the IRB-BCH granted a waiver for informed consent. This exemption, based on the provisions of Resolution 466/2012, took into account the potential emotional burden that contacting surviving family members might cause. Furthermore, as a retrospective study, the research was restricted to analyzing pre-existing slides and paraffin-embedded tissue blocks stored in the hospital’s pathology department, along with reviewing medical records.

### 2.2 DNA isolation

Genomic DNA was extracted from both frozen tumor tissue and buffy coat with the QIAsimphony DNA Mini Kit (Qiagen) on the QIAsimphony semi-automated platform (Qiagen), following the institution’s standardized protocol (16). DNA quantity and quality were assessed by Qubit (Life Technologies) and TapeStation Systems (Agilent).

### 2.3 Whole-exome sequencing

Whole-exome sequencing (WES) was conducted by SOPHiA Genetics (SOPHiA Genetics SA, Rolle, Switzerland) using an Illumina NovaSeq sequencer (Illumina, San Diego, CA, USA). The SOPHiA Whole Exome Solution Kit (version 1) was employed, covering 203,058 target regions, encompassing 40,907,213 base pairs across 19,682 genes. Tumor sample sequencing achieved a mean coverage of 75x across 90% of the analyzed regions.

Alignment to the human genome (version hs37d5-decoy, build 37) was performed using Burrows-Wheeler Aligner (version 0.7.10-r789) (17), followed by removal of duplicate reads using Picard-Tools 1.92 (http://broadinstitute.github.io/picard/) (18).

Somatic variant calling (single nucleotide variants (SNVs) and insertions/deletions) was performed using MuTect2 (https://gatk.broadinstitute.org/hc/en-us/articles/360036485152-Mutect2) (18) and PINDEL version 0.2.5t (http://gmt.genome.wustl.edu/packages/pindel/) (19).

Germline variants were filtered using the gnomAD v. 2.1.1 database (https://gnomad.broadinstitute.org/) and a pool of normal samples from 291 patients from Barretos Cancer Hospital. A variant allele frequency (VAF) threshold of 10% and a sequencing depth of 50x were applied post-annotation (20).

For functional annotation and consequence analysis, we employed ANNOVAR (21) and excluded all variants considered benign or likely benign by the ClinVar (22) or InterVar (23) databases. We considered candidate variants all those called pathogenic or likely pathogenic according to ClinVar and those known or predicted as oncogenic or driver by the Cancer Genome Interpreter (CGI) tool (24). All candidate variants were visually confirmed using Integrated Genomics Viewer (IGV) (25). The SigProfiler Assignment (26, 27) tool was employed for identification of mutation profiles and tumor mutation burden based on the somatic mutations found within our cohort. The profiles were then correlated with the signatures described by the Catalogue of Somatic Mutations in Cancer (COSMIC) (28). Nexus Copy Number software (BioDiscovery Inc., El Segundo, CA) (http://www.biodiscovery.com/nexus-copy-number/) was used for the DNA copy number alterations (CNA) analysis. The log2 ratio of the tumor/control sample intensities was calculated, and altered regions were identified using Fast Adaptive States Segmentation Technique 2 (FASST2). Regions of interest were further classified using the Cancer Gene Census (CGC) database (https://cancer.sanger.ac.uk/census) and analyzed with Significance Testing for Aberrant Copy Number – STAC (http://cbil.upenn.edu/STAC) (29). Copy-number variations with 100% overlap were excluded using the Database of Genomic Variants – DGV (http://dgv.tcag.ca/dgv/app/home) (25).

### 2.4 Validation of somatic variants

Validation of somatic variants identified in WES was conducted through bidirectional Sanger sequencing for the genes *KIT*, *KRAS*, and *MTOR*, targeting specific mutations with corresponding primer sets: *KIT* Ala829Pro (Forward: “CATTTCAGCAACAGCAGCATCT”; Reverse: “CACAAGGAAGCAGGACACCAA”), and *KRAS* Gln61Leu (Forward: “CAGGATTCCTACAGGAAGCAAGTAG”; Reverse: “CACAAAGAAAGCCCTCCCCA”).

Genomic DNA from the patients underwent polymerase chain reaction (PCR) amplification and subsequent purification using the ExoSAP-IT™ enzyme (Thermo Fisher Scientific). Sanger sequencing reaction was carried out utilizing BigDye Terminator kit (Thermo Fisher Scientific), followed by purification with the BigDye X-terminator kit (Thermo Fisher Scientific), and sequencing on the automated sequencer ABI 3500XL (Applied Biosystem). Additionally, the Illumina panel TruSight Tumor 15 was employed to validate the *KIT* mutation (p.Asp816Val) following the protocol as previously described (30). For library preparation, targets were enriched via multiplex PCR and verified by electrophoresis. Paired-end sequencing was then performed using the MiSeq Reagent Kit v3 (600 cycles) on the Illumina MiSeq platform.

## 3 Results

### 3.1 Clinicopathologic features of patients

The clinicopathologic features of the 16 pediatric GCT patients are summarized in **Table 1**. Among the 16 patients analyzed, the majority presented with ovarian tumors (n = 10, 62.5%), while five (31.2%) had testicular tumors, and one (6.3%) had mediastinal tumor. The mean age at diagnosis was 11.4 years (range, 2–18 years), and most patients were female (68.8%). Clinical findings showed that eight (50%) patients had mixed GCTs, followed by three (18.8%) with yolk sac tumor, three (18.8%) with mature teratoma, one (6.3%) with embryonal carcinoma, and one (6.3%) with dysgerminoma. More than half of the patients were classified as low-risk (n = 9, 56.3%), and 25% (n = 4) had metastases at diagnosis. Therefore, four patients (GCT-03, GCT-11, GCT-18, GCT-22) had tumor recurrence (25%), and 93.7% of patients were still alive and without cancer at the time of analysis.

**Table 1:** Clinicopathologic features of pediatric patients with GCT.

Features	Total	Ovary	Testis	Mediastinum
**N (%)**	16 (100%)	10 (62.5%)	5 (31.2%)	1 (6.3%)
Average Age (Years)				
2–9 years	6 (37.5%)	3 (50%)	2 (33%)	1 (17%)
13–18 years	10 (62.5%)	7 (70%)	3 (30%)	–
Gender				
Female	11 (68.8%)	10 (100%)	–	1 (100%)
Male	5 (31.2%)	–	5 (100%)	–
Histology				
Yolk Sac Tumor	3 (18.8%)	1 (10%)	2 (40%)	–
Embryonal Carcinoma	1 (6.3%)	1 (10%)	–	–
Mature Teratoma	3 (18.8%)	2 (20%)	–	1 (100%)
Mixed GCT	8 (50%)	5 (50%)	3 (60%)	–
Dysgerminoma	1 (6.3%)	1 (10%)	–	–
Risk				
Low	9 (56.3%)	6 (60%)	2 (40%)	1 (100%)
Intermediate	1 (6.3%)	1 (10%)	–	–
High	6 (37.5%)	3 (30%)	3 (60%)	–
Metastasis				
Yes No	4 (25%) 12 (75%)	1 (10%) 9 (90%)	3 (60%) 2 (40%)	–
				1 (100%)
Relapse				
Yes No	4 (25%) 12 (75%)	3 (30%) 7 (70%)	1 (20%) 4 (80%)	–
				1 (100%)
Status				
Disease-free survival	15 (93.7%)	10 (100%)	5 (100%)	–
Death of disease	1 (6.3%)	-	–	1 (100%)

All metastatic cases involved pulmonary dissemination (4/4), with additional sites such as liver or lymph nodes in a subset of patients. Notably, all tumors presenting metastasis contained a yolk sac tumor (YST) component, either as pure YST or within a mixed histology.

### 3.2 Mutational profile

#### 3.2.1 Single nucleotide variants

**Figure 1:**
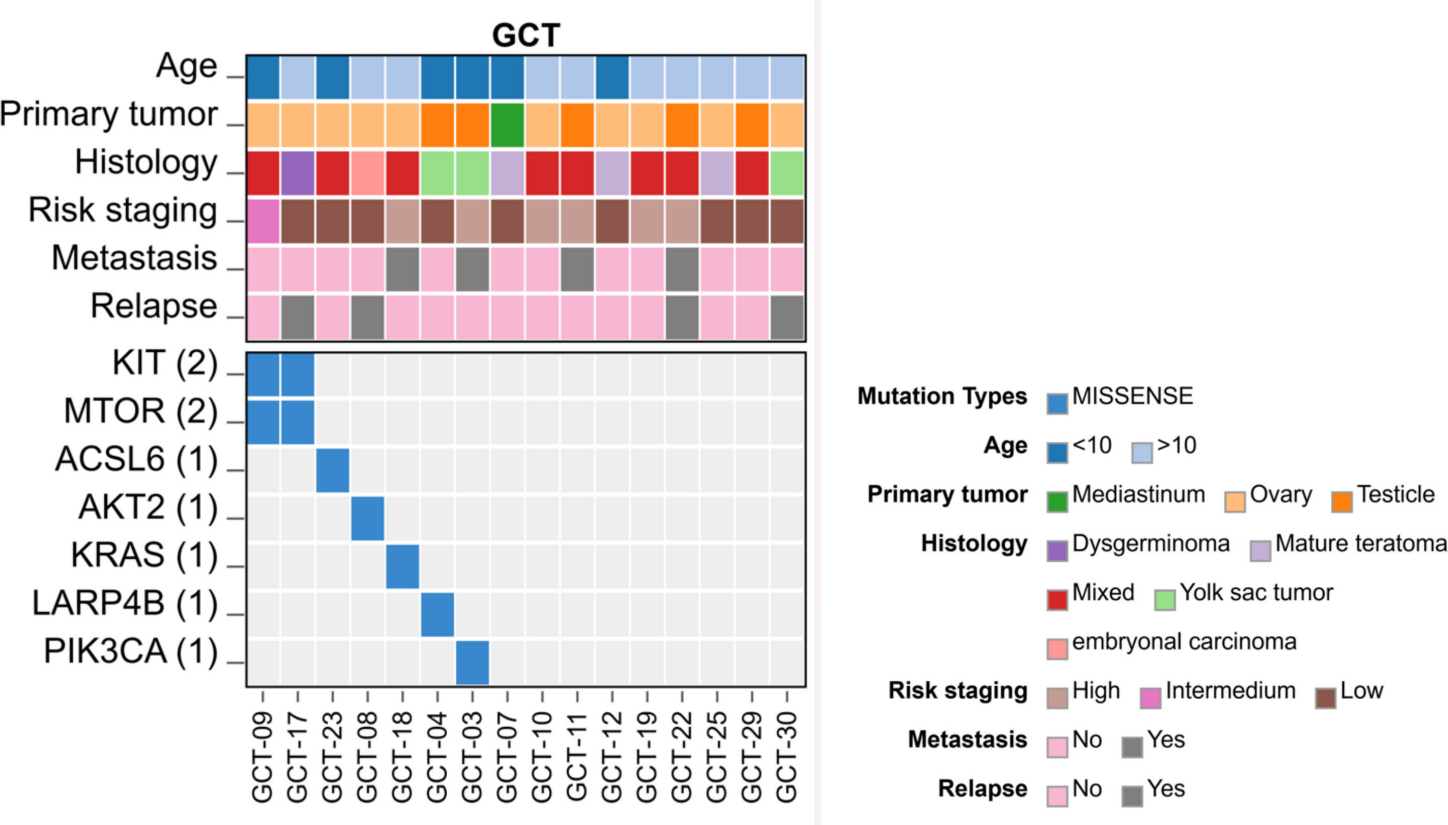
Heatmap illustrating Single Nucleotide Variants (SNVs) in pediatric GCTs, categorized by age, primary site, histology, staging, metastasis and relapse status (n = 16).

We evaluated the landscape of somatic clinically actionable driver alterations. Several variants were detected (**Supplementary Table S1**); however, only seven were predicted or annotated as oncogenic, according to ClinVar and CGI (**Table 2**, **Figure 1**).

**Table 2:** Molecular data on driver genes identified from the whole-exome sequencing of pediatric patients with germ cell tumors.

Chr	Gene	Position	Reference allele	Altered allele	Transcript	Exon	c.DNA	Protein	VAF	Depth	Oncogenicity	CGI
Patient GCT_03												
3	*PIK3CA*	178938877	G	C	NM_006218	14	c.G2119C	p.E707Q	16.0%	160	oncogenic (predicted)	driver (boostDM)
Patient GCT_04												
10	*LARP4B*	888905	T	G	NM_015155	7	c.A613C	p.T205P	18.0%	178	oncogenic (predicted)	driver (oncodriveMUT)
Patient GCT_08												
19	*AKT2*	40742001	T	C	NM_001626	11	c.A971G	p.D324G	29.1%	300	oncogenic (predicted)	driver (oncodriveMUT)
Patient GCT_09												
1	*MTOR*	11168338	C	G	NM_004958	57	c.G7534C	p.D2512H	22.9%	147	oncogenic (predicted and annotated)	driver (oncodriveMUT)
4	*KIT*	55599321	A	T	NM_000222	17	c.A2447T	p.D816V	21.5%	120	oncogenic (predicted and annotated)	driver (boostDM)
Patient GCT_17												
1	*MTOR*	11194411	G	A	NM_004958	37	c.C5243T	p.A1748V	30.2%	192	oncogenic (predicted)	driver (oncodriveMUT)
4	*KIT*	55602664	G	C	NM_000222	18	c.G2485C	p.A829P	30.1%	101	oncogenic (predicted and annotated)	driver (boostDM)
Patient GCT_18												
12	*KRAS*	25380276	T	A	NM_004985	3	c.A182T	p.Q61L	44.5%	231	oncogenic (predicted and annotated)	driver (boostDM)
Patient GCT_23												
5	*ACSL6*	131307317	G	A	NM_015256	14	c.C1360T	p.R454W	29.7%	257	oncogenic (predicted)	driver (oncodriveMUT)

Chr, Chromosome; c.DNA, Complementary DNA; VAF, Variant allele frequency; CGI, Cancer Genome Interpreter.

The genes with variants in more than one sample were *MTOR* and *KIT*, showing missense mutations in two ovarian GCTs (GCT-09 and GCT-17). Patient GCT-09 was diagnosed with a mixed GCT in the left ovary at the age of six, staged as FIGO-IIC (intermediate risk), with no occurrence of metastasis or relapse, while patient GCT-17 was diagnosed with dysgerminoma of the right ovary at the age of 13, staged as FIGO-IA (low risk), with no occurrence of metastases. After one year, the patient experienced recurrence in the retroperitoneal lymph node (**Supplementary Table S1**).

The *MTOR* variant p.Asp2512His (c.G7534C), situated in exon 57 (GCT-09), is classified by ClinVar as level 3 with potential clinical significance among solid tumors. The other missense mutation observed in *MTOR* identified in sample GCT-17 (p.Ala1748Val - c.C5243T) in exon 37, affecting the rapamycin-binding domain of the protein. The *KIT* variant detected in the GCT-09 patient was p.Asp816Val (c.A2447T) in exon 17. GCT-17 patient harbors the p.Ala829Pro (c.G2485C) variant in exon 18 of the gene.

Patient GCT-18, diagnosed with mixed GCT, exhibited a missense mutation (p.Gln61Leu - c.A182T) in exon 3 of the *KRAS* oncogene, alongside a gain in the number of copies of this gene. This patient was diagnosed at age 15 with stage FIGO-IV (high risk) and presented with lung metastasis. In total, four variants annotated as predicted driver events were identified in the cohort (*KIT* p.Asp816Val, *KIT* p.Ala829Pro, *MTOR* p.Asp2512His, *KRAS* p.Gln61Leu) and all of them occurred in ovarian tumors and contained a dysgerminoma component.

Gene *PIK3CA* showed a variant in patient GCT-03, who was diagnosed with a pure yolk sac tumor of the testis at the age of 2 and had lung metastasis, which classified him as a high-risk stage COG-IV patient. Patient GCT-08, diagnosed at 15 years old with an embryonal carcinoma of the ovary and no metastasis, had a missense alteration in gene *AKT2*. Despite being classified as stage I and low risk, this patient had a relapse in the ovary.

The other two alterations in our cohort were in the genes *LARP4B* and *ACSL6* in patients GCT-04 and GCT-23, respectively. Patient GCT-04 was a 2-year-old toddler with a yolk sac tumor of the test is classified as stage COG-I and low risk, while patient GCT-23 was a 3-year-old girl with a mixed tumor of the ovary, also classified as stage FIGO-I and low risk.

#### 3.2.2 Validation of somatic variants

**Figure 2:**
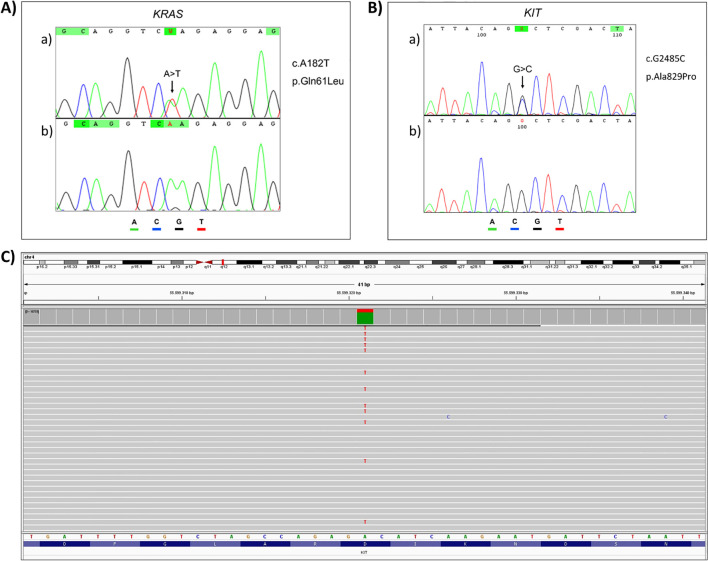
Analysis of *KRAS* and *KIT* variants. Validation of **(A)***KRAS* (c.A182T; p.Gln61Leu) and **(B)***KIT* (c.G2485C; p.Ala829Pro) variants using Sanger sequencing. a) Tumor sample. b) Normal sample. **(C)** Validation of *KIT* (c.A2447T; p.Asp816Val) using TruSight Tumor 15 panel.

Selected variants were validated through conventional sequencing methods. The missense mutation of *KRAS* (p.Gln61Leu), detected in an ovarian mixed tumor (GCT-18) with a VAF = 44.5%, was confirmed by Sanger sequencing (**Figure 2A**).

The *KIT* gene exhibited two missense mutations in two distinct patients. The variant p.Ala829Pro, found in an ovary dysgerminoma sample (GCT-17), with VAF = 30.1%, was validated through Sanger sequencing (**Figure 2B**).

The second *KIT* mutation identified was p.Asp816Val in an ovary-mixed tumor (GCT-09) with VAF = 21.5%, which was validated using the TruSight Tumor 15 panel (Illumina) (**Figure 2C**).

#### 3.2.3 Tumor mutational burden

**Figure 3:**
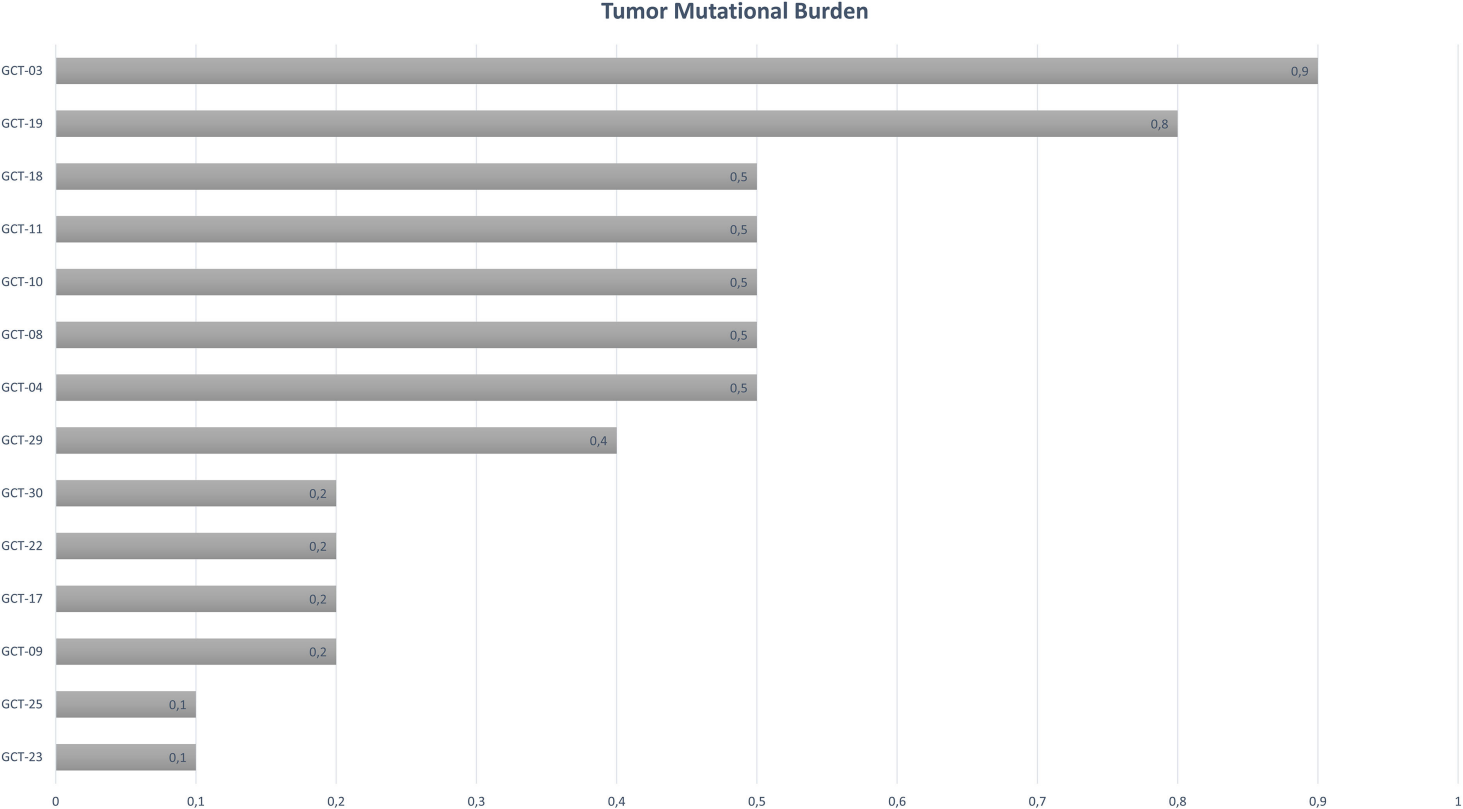
Somatic mutation frequency (mutations/Mb) from exome sequencing (n = 14).

The Tumor Mutational Burden (TMB) is a quantitative measure of somatic mutations found in each sample. TMB values above 0.1 mutations per megabase (mut/Mb) were found in all malignant tumor samples (n = 14, 87.5%). GCT-07 and GCT-12 are benign mature teratomas and had a mutation frequency below 0.1 mut/Mb, and were therefore not included in the analysis. The overall median frequency among the fourteen samples was 0.4 mut/Mb, with a range of 0.1 to 0.9 (mut/Mb) (**Figure 3**).

The highest TMB values (0.9 mut/Mb and 0.8 mut/Mb) were identified in samples GCT-03 and GCT-19, respectively. Both were from patients with high-risk staging and predominantly yolk sac tumor histology. Sample GCT-3 is a testicular YST (COG IV) from a 2-year-old patient with lung metastases and no recurrence, which harbored a PIK3CA variant. Sample GCT-19 is a mixed ovarian GCT (>90% YST) (FIGO III) from a 13-year-old patient with no metastases and no recurrence (**Supplementary Table S1**).

#### 3.2.4 Single base substitution signatures

**Figure 4:**
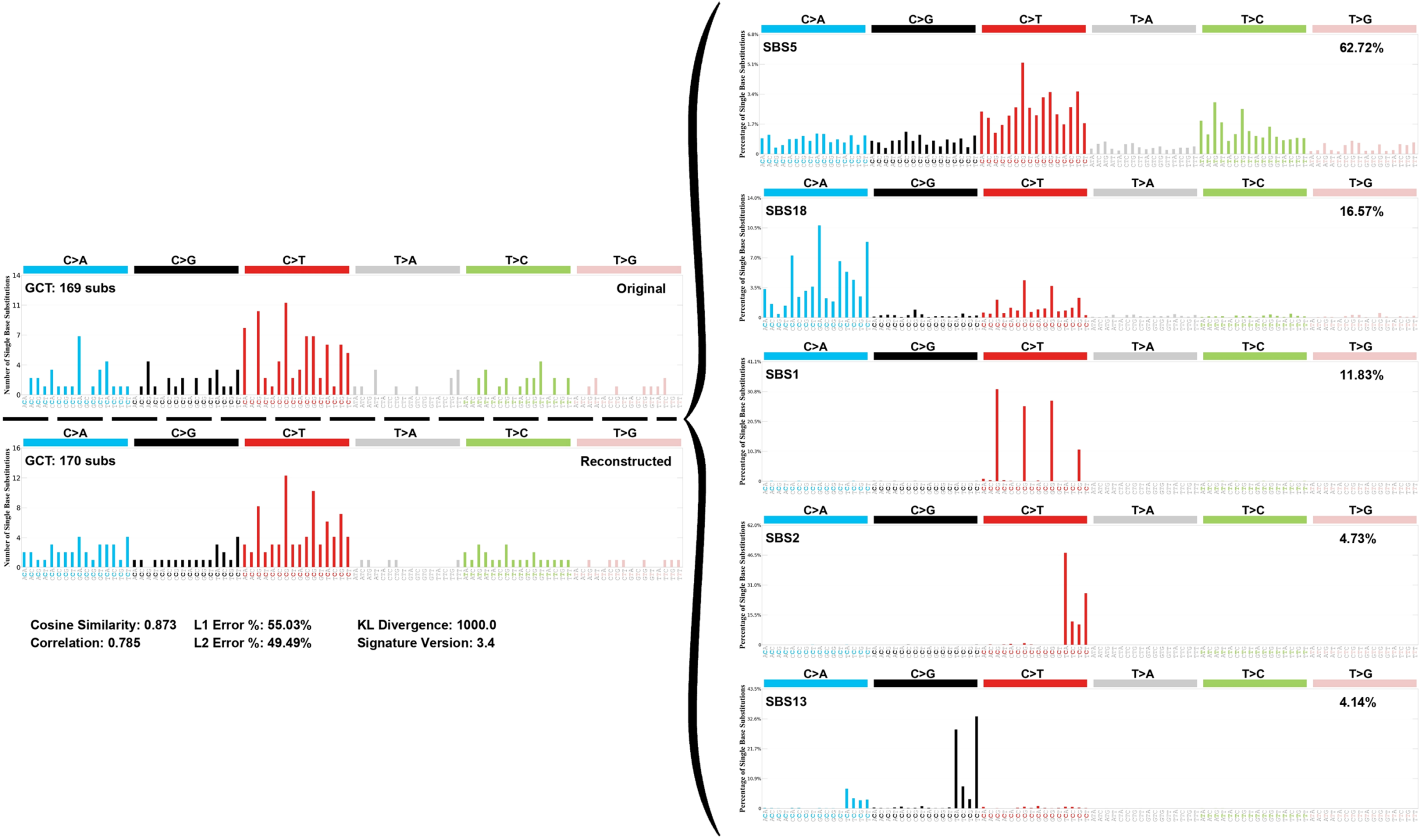
Mutational signature decomposition of 16 pediatric germ cell tumors.

Due to the low mutation rate in our samples, individual signatures could not be assessed, however, by aggregating the detected alterations we identified a profile of somatic single base substitutions for our cohort, which included SBS1, SBS2, SBS5, SBS13, and SBS18 (**Figure 4**).

Our reconstructed mutational profile closely matched the observed mutation pattern, achieving a cosine similarity of 0.873 and a correlation of 0.785, indicating a strong agreement between the original and reconstructed profiles.

Among the identified signatures, SBS5 was the most prevalent, contributing 62.72% of all detected SBS mutations, followed by SBS18 (16.57%), SBS1 (11.83%), SBS2 (4.73%), and SBS13 (4.14%).

### 3.3 Analysis of copy number alterations

**Figure 5:**
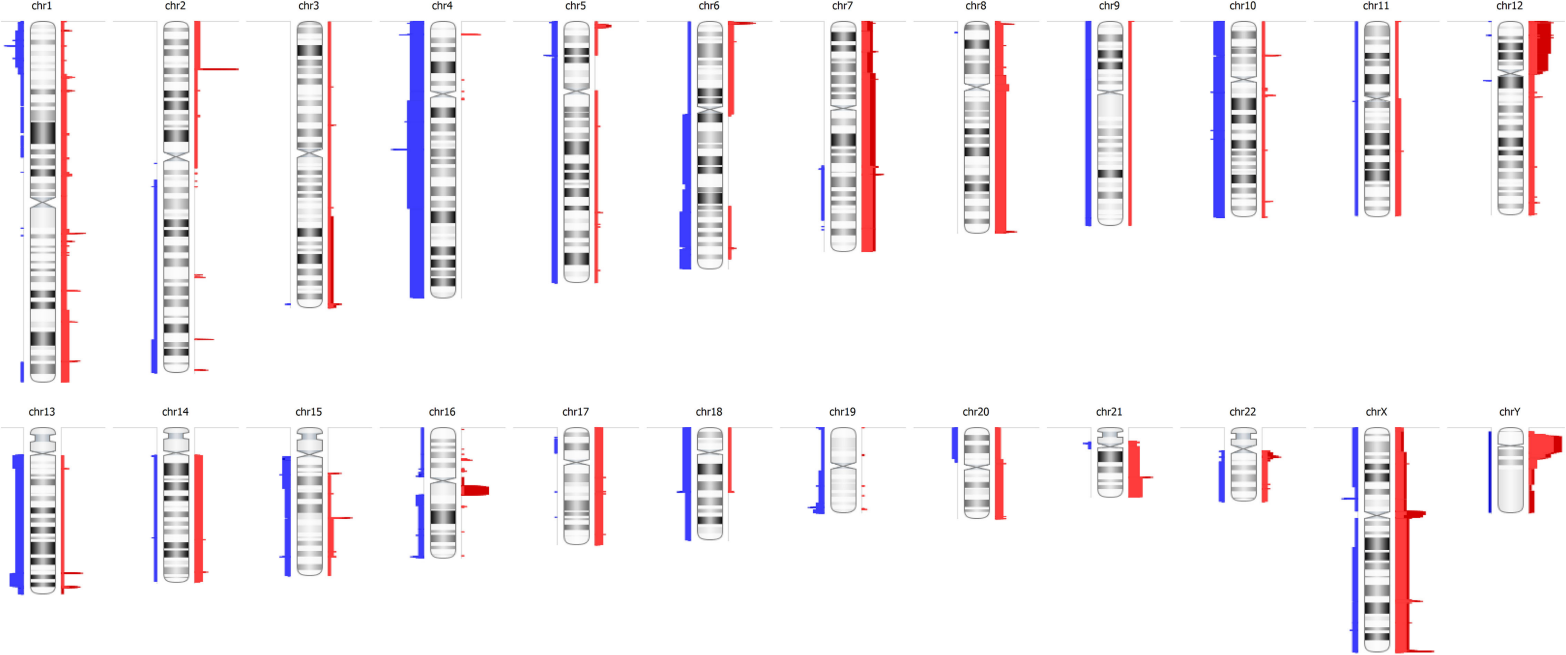
Visualization of genome-wide copy number variations of all patients, comprising deletions and amplifications, at the chromosome level. Chromosome numbers are indicated in black. Blue and red regions represent deletion and amplification, respectively.

We initially investigated the copy number alterations (CNAs), showing a higher amplification frequency in regions of chromosomes 7, 8, 12, 21, and 22 and deletions in regions of chromosomes 4 and 10 (**Figure 5**).

After the 25% minimum frequency threshold, 58 events of CNAs were identified, including 44 (75.86%) amplifications and 14 (24.14%) deletions.

CNAs were detected on chromosome four within two primary regions (p16.3-p16.1 and p16.1-q35.1), as per the CGC. These regions encompass the oncogenes *FGFR3*, *PDGFRA*, *KIT*, and *FBXW7*, with all significant alterations manifesting as copy number losses. Among the samples, six exhibited losses in chromosome four (37.5%), comprising three testicular tumors, being two mixed GCTs (GCT-11 and GCT-22), and one yolk sac tumor (GCT-04). Additionally, three ovarian samples displayed losses, with two being mixed GCTs (GCT-10 and GCT-18) and one being embryonal carcinoma (GCT-08). Notably, 66% of samples displaying losses in chromosome four were associated with mixed GCTs. Among the six samples exhibiting losses in chromosome 4 (37.5%), deficiencies were observed in the *KIT* and *PDGFRA* genes. Within this subset, five samples (GCT-08, GCT-10, GCT-11, GCT-18, and GCT-22) demonstrated deletions affecting *FBXW7* and *FGFR3* (31.25%). Four out of these five samples were mixed GCTs (80%). Interestingly, none of the mature teratoma samples displayed notable aberrations in the copy number of these genes.

Chromosome 7 exhibited notable gains in two primary regions (p15.3-q22.3 and q22.3-q36.3). The first region encompasses the *EGFR* gene, while the second includes the *BRAF* gene. Amplification of both genes occurred concurrently in five samples (GCT-10, GCT-11, GCT-17, GCT-19, and GCT-29), constituting 31.25% of the total samples. Among these, three samples originated from the ovary and two from the testis, with four classified as mixed GCTs (80%) and one as ovarian dysgerminoma (20%). Among the mixed GCTs, two were ovarian and two were testicular.

Significant changes on chromosome 8 manifested as gains in the *FGFR1* and *MYC* genes within the p11.23-q24.3 region. Four samples (25%) exhibited simultaneous amplifications in both genes (GCT-09, GCT-10, GCT-22, and GCT-29), all of which were mixed GCTs.

Chromosome 10 had a loss in the copy number of the *PTEN*, *NFKB2*, *SUFU*, and *FGFR2* genes within the q22.3-q26.3 region. Four samples (GCT-08, GCT-10, GCT-11, and GCT-22) exhibited concurrent deletions in all four genes, accounting for 25% of the samples. Among these, three (75%) were mixed GCTs. Additionally, seven samples showed a gain in *KRAS*, with six of them (85.7%) also classified as mixed GCTs.

All alterations on chromosome 12 were amplifications, with five genes, *KRAS*, *CCND2*, *KDM5A*, *ZNF384*, and *ETV6*, located in the 12p13.33-q11 region. Fifty percent of patients (n = 8 - GCT-03, GCT-08, GCT-09, GCT-10, GCT-11, GCT-18, GCT-22, and GCT-29) showed gains in chromosome 12. All exhibited amplification of the *CCND2*, *KDM5A*, and *ETV6* genes, with six samples being mixed GCTs, representing 75% of the eight samples. Seven samples showed gain in *KRAS*, six of which (85.7%) were mixed GCTs. Four mixed GCTs patients were confirmed to have two or more copies of *KRAS*, *CCND2*, *ETV6*, and *KDM5A*. None of the mature teratoma samples exhibited significant alterations in the copy number of chromosome 12 regions.

Chromosome 21 exhibited amplifications in the largest number of samples (n = 9, 56.25% - GCT-04, GCT-07, GCT-08, GCT-09, GCT-10, GCT-11, GCT-17, GCT-22, and GCT-29). The predominant region encompasses the *OLIG2* and *ERG* genes (p11.1-q22.3). The *OLIG2* gene experienced copy number gains in all nine samples, comprising four ovarian GCTs, four testicular, and one mediastinal sample. Among these, five samples were mixed GCTs (55.5%). The sole sample of mature mediastinal teratoma in the study exhibited amplification of the *OLIG2* gene, with two or more copies gained, and no other significant copy number changes were noted in this sample.

Chromosome 22 displayed copy number gain alterations in two primary regions (q11.1-q11.21: *CLTCL1* and q11.21: *BCR*). Four samples (25% - GCT-03, GCT-04, GCT-10, and GCT-19) exhibited amplification of both genes. Among these samples, 50% were classified as mixed ovarian GCTs, while the remaining 50% were testicular yolk sac tumors. All CNAs are summarized in **Table 3**.

**Table 3:** Copy number alteration in pediatric GCT patients by chromosomal region.

Chromosomal location	Region size	Cytobands	Event	Genes	CGC
chr4:34,732-8,745,154	8710422	p16.3 - p16.1	Loss	129	*FGFR3*, *WHSC1*
chr4:9,269,936-190,926,232	181656296	p16.1 - q35.2	Loss	1005	*SLC34A2*, *PHOX2B*, *FIP1L1*, *PDGFRA*, *CHIC2*, *KIT*, *KDR*, *RAP1GDS1*, *TET2*, *IL2*, *FBXW7*
chr6:132,045,489-155,757,066	23711577	q23.2 - q25.3	Loss	173	*MYB*, *TNFAIP3, ECT2L*
chr6:157,355,483-161,029,352	3673869	q25.3 - q26	Loss	43	*EZR*
chr6:161,102,668-170,948,848	9846180	q26 - q27	Loss	80	*FGFR1OP*, *MLLT4*
chr7:1,477,626-6,661,400	5183774	p22.3 - p22.1	Gain	75	*CARD11*, *PMS2*
chr7:6,730,899-20,776,600	14045701	p22.1 - p21.1	Gain	65	*ETV1*
chr7:21,493,105-105,706,361	84213256	p15.3 - q22.3	Gain	776	*HNRNPA2B1*, *HOXA9*, *HOXA11*, *HOXA13*, *JAZF1*, *IKZF1*, *EGFR*, *SBDS*, *ELN*, *HIP1*, *AKAP9*, *CDK6*
chr7:105,737,172-158,912,527	53175355	q22.3 - q36.3	Gain	455	*MET*, *SMO*, *CREB3L2*, *KIAA1549*, *BRAF*, *EZH2*, *MLL3*
chr8:37,593,920-145,318,628	107724708	p11.23 - q24.3	Gain	676	*WHSC1L1*, *FGFR1*, *HOOK3*, *TCEA1*, *PLAG1*, *CHCHD7*, *NCOA2*, *HEY1*, *COX6C*, *EXT1*, *MYC*, *NDRG1*
chr8:145,534,417-146,225,143	690726	q24.3	Gain	41	*RECQL4*
chr10:80,757-23,309,986	23229229	p15.3 - p12.2	Loss	184	*GATA3*, *MLLT10*
chr10:24,615,220-49,319,950	24704730	p12.1 - q11.22	Loss	189	*KIF5B*, *RET*
chr10:49,368,523-81,471,704	32103181	q11.22 - q22.3	Loss	234	*NCOA4*, *CCDC6*, *PRF1*
chr10:81,656,192-135,438,885	53782693	q22.3 - q26.3	Loss	513	*BMPR1A*, *FAM22A*, *PTEN*, *TLX1*, *NFKB2*, *SUFU*, *VTI1A*, *TCF7L2*, *FGFR2*
chr12:133,425-36,445,147	36311722	p13.33 - q11	Gain	388	*KDM5A*, *CCND2*, *ZNF384*, *ETV6*, *KRAS*
chr13:100,828,141-109,766,480	8938339	q32.3 - q33.3	Loss	41	*ERCC5*
chr21:12,935,265-48,080,340	35145075	p11.1 - q22.3	Gain	373	*OLIG2*, *RUNX1*, *ERG*, *TMPRSS2*, *U2AF1*
chr22:17,055,191-20,323,202	3268011	q11.1 - q11.21	Gain	84	*CLTCL1*
chr22:20,459,035-21,922,280	1463245	q11.21	Gain	41	*BCR*

CGC, Cancer Gene Census-Sanger.

## 4 Discussion

Genomics studies on GCTs have primarily focused on adult patients, leaving a significant gap in our understanding of pediatric GCTs. The rarity and heterogeneity of these tumors present challenges, including limited sample availability and variations in behaviors among histological subtypes, hindering comparative analysis. One of the main goals of our study was the identification of genes potentially driving the malignancies in pediatric GCTs. Mutations in *KIT* were found in two samples (12.5%). *KIT* is expressed by different cell types, and once activated, it triggers the activation of proteins, thereby triggering different signaling pathways, notably *MAPK*/*MEK*, *PI3K*/*AKT*, and *JAK*/*STAT* (31). In addition, *KIT* mutation has been associated with the sensitivity of testicular GCTs to platinum-based treatment and is also present in ovarian GCTs with dysgerminoma components (32–35). Orthogonal methodologies validated both *KIT* mutations.

The *KIT*-Asp816 codon is classified as a hotspot, and mutations in this region are associated with various tumor types, like acute myeloid leukemia (36), gastrointestinal stromal tumors (37), and systemic mastocytosis (15) and have been studied as target for specific treatments. Chlorpromazine is an antipsychotic drug that leads to cell death in AML cells *in vitro* and *in vivo* by interfering with the intracellular localization of *KIT-D816V* (38). This mutation is also the target of the drug avapritinib, a new and selective type 1 *KIT* inhibitor that focuses on D816V (14, 39). This medication is FDA-approved for patients with systemic mastocytosis and unresectable or metastatic gastrointestinal stromal tumor (14, 40). In patients with systemic mastocytosis, the phase 1 EXPLORER trial showed the drug induced deep and durable responses, including the molecular remission of the mutation (41). These results show this mutation holds promise as a potential therapeutic target for germ cell tumors as well.

One patient with an ovarian mixed tumor (GCT-18) and lung metastasis showed a missense mutation (p.Gln61Leu) in the *KRAS* gene, along with its amplification. The Gln61 codon is classified as a hotspot in various molecular databases, and mutations in this region or amplifications promote the gain of gene function (TCGA, COSMIC, OncoKB). Besides promoting gain of function, this gene has been identified in studies of metastatic tumors (42), testicular, and ovarian GCTs (6, 33, 43). Our previous study also showed *KRAS* mutations and amplification in adult testicular GCTs (30), highlighting this gene’s relevance for targeted approaches in TGCT. In addition, alterations in *KRAS* have been studied as a potential drug target for solid tumors with the G12C alteration (44). The drugs adagrasib and sotorasib are both *KRAS*-G12C inhibitors with ongoing clinical trials. Sotorasib showed encouraging anticancer activity in the phase 1 clinical trial in a cohort comprising non-small-cell lung cancer (NSCLC), colorectal cancer, and other solid tumors (12). When compared with standard-of-care treatment docetaxel for NSCLC in a phase 3 trial, it increased progression-free survival and showed a safer profile (45). Similarly, a phase 1 trial (KRISTAL-1) treated 25 patients with adagrasib, which achieved good tolerance and showed antitumor activity, leading to phase 3 trials that are currently ongoing (13). For many years, *KRAS* mutations were considered undruggable, however, recent progress in bioengineering, organic chemistry, and allied fields has provided a robust foundation for the direct targeting of *KRAS* (44), especially the G12C mutation as mentioned above. Other mutations are also being studied with pan-*KRAS* inhibitors that will have broader therapeutic implications (46).

*MTOR* mutations were also found in two samples (12.5%). *MTOR* is classified as a cancer driver and recognized as an oncogene implicated in GCT development. The signaling pathway PI3K/AKT/mTOR is one of the main growth regulation pathways in normal and cancer cells, which is activated by tyrosine kinase receptors that phosphorylate different members of the pathway, like PI3K, AKT, mTOR, and PTEN (47).

Although the variant found in the *PIK3CA* gene was only reported once as associated with triple-negative breast cancer (48), this gene is a main part of the signaling pathway PI3K/AKT/mTOR, which has been reported to be altered in some cancer types and it is usually used as a prognostic marker and therapeutic target (49–52). Also, part of this important pathway is gene *AKT2*, which plays a pivotal role in many malignancies by having alterations that frequently hyperactivate its protein kinase (53–55). However, despite being predicted as oncogenic, the variant found in patient GCT-08 has not been elucidated.

While some of the alterations identified in *KIT*, *KRAS*, *MTOR*, *PIK3CA*, and *AKT2* intersect with signaling pathways that have been explored as therapeutic targets in other solid tumors, it is important to note that, apart from the canonical *KIT* D816V hotspot, the majority of variants detected in our cohort represent predicted or previously uncharacterized alterations. Although the *MTOR* (p.A1748V and p.D2512H), *PIK3CA* (p.E707Q), and *AKT2* (p.D324G) variants were annotated in ClinVar or predicted as oncogenic drivers by the Cancer Genome Interpreter, these alterations are rare and lack experimental validation. *MTOR* p.A1748V and p.D2512H have been reported infrequently in public databases and may represent variants of uncertain significance, and similarly, *PIK3CA* p.E707Q and *AKT2* p.D324G have no established pathogenicity. Therefore, despite involving genes that participate in canonical PI3K/AKT/mTOR signaling, the biological and clinical relevance of these specific variants in pediatric GCTs remains unknown. Targeted therapies are not part of standard management for pediatric GCTs, given their typically high chemosensitivity (5), and any translational implications of these findings remain theoretical at this stage. Functional studies will be essential to clarify whether these alterations contribute to tumor behavior or could inform future precision-oncology strategies in rare refractory or metastatic cases.

Recent genomic studies further support the molecular patterns observed in our cohort. Several groups have demonstrated that germ cell tumors segregate primarily by histological subtype rather than gonadal site, including the seminal comparative analyses by Kraggerud et al. (2013) and subsequent reports by Shen et al. (2018) and Chovanec et al. (2018). More recent literature has refined this framework: a comprehensive review of adolescent and young adult GCTs emphasized recurrent *KRAS* alterations and PI3K/AKT pathway events in yolk sac tumors, and *KIT* mutations as defining lesions in dysgerminomas (56). These observations are in agreement with the genomic characterization by Van Nieuwenhuysen et al. (2018), who showed that ovarian dysgerminomas frequently harbor activating *KIT* mutations and other tyrosine kinase–related alterations (57).

Our results are consistent with these subtype-specific signatures. As identified in our cohort, all predicted and annotated oncogenic variants (*KIT* p.Asp816Val, *KIT* p.Ala829Pro, *MTOR* p.Asp2512His, and *KRAS* p.Gln61Leu) occurred exclusively in ovarian tumors containing a dysgerminoma component. Likewise, the cases presenting metastatic disease uniformly involved tumors with a yolk sac tumor (YST) component and exhibited focal chromosome 12p amplification involving *KRAS*, *CCND2*, *KDM5A*, and *ETV6*, a hallmark alteration also widely associated with aggressive GCT behavior in the literature. Together, these findings reinforce that, even within a small pediatric cohort, the genomic profiles observed align with well-established histology-driven molecular patterns described across larger adult and pediatric studies, including those reported in the PeCan cohort from Saint Jude Children’s Research Hospital (58).

Within our cohort’s mutational landscape, we also assessed the tumor mutational burden. Pan-cancer analyses of a broad spectrum of tumors have shown that the mutational burden of pediatric cancers (0.02–0.49 mut/Mb) is more than 10-fold lower than the TMB of adults (0.13–1.8 mut/Mb) (8, 59, 60). The average TMB values ​​found in this work were 0.4 mut/Mb, consistent with those previously reported for GCTs, particularly in the pediatric population (6, 32, 61). While elevated TMB in GCTs has been proposed as a potential factor in treatment resistance (32), our high-risk patients with higher TMB exhibited a complete response to therapy. Additionally, recent studies have demonstrated the potential of TMB as a biomarker for response to immune checkpoint inhibitors, due to the formation of tumor-specific neoantigens (62).

Single base mutational signatures were assessed, revealing the presence of SBS18, which is known to be related to oxidative DNA damage and has been reported to be prevalent in other childhood cancer types like neuroblastoma and rhabdomyosarcoma (7, 8). SBS1, a common signature across various tumor types, is attributed to spontaneous deamination events and reflects the accumulation of mutations with cell division (27). Additionally, SBS2 and SBS13, which are correlated and were also identified in our samples, have been linked to chromothripsis and *TP53* mutations in pediatric tumors (8).

In addition to somatic mutations, CNAs are well-established molecular features in adult GCTs and recent studies of pediatric GCTs (63). In the current study, 58 significant events (frequency ≥25%) of gene gains and losses were observed, with 44 amplifications and 24 deletions. Gain of the short arm of chromosome 12 (12p) is a universal feature among adult testicular GCTs, as well as copy number gains on chromosomes 7, 8, 21, 22, and X (6, 64). Recently, Loveday et al. (2020) analyzed copy number alterations in 188 adults testicular GCT samples using whole exome sequencing. The most frequent events occurring in chromosomal arms were gain of 12p, 21q, and 7p; in addition to focal amplifications at 12p13.32, 12p12.1, and 12p11.21 (32). These findings corroborate our data, in which focal amplifications on chromosome 12 were observed in seven samples, six of which were mixed GCTs. Among the significant events on chromosome 12, gains involving the *KRAS* and *CCND2* genes were particularly important, due to their high frequency within our cohort and their previous correlation with the development of GCTs (6, 63).

Although we obtained interesting new findings, there are limitations to the present study. First, the sample size was relatively small, which may impact the generalizability of the results. Our cohort did not include CNS germ cell tumors, as these cases are managed through separate neuro-oncology referral pathways in Brazil and were therefore not represented among the available samples. Additionally, the histological subtypes of germ cell tumors were underrepresented in our sample, potentially limiting the applicability of our findings across all subtypes. Moreover, while the mutations identified in this study were predicted to be disease-causing variants, their precise functional significance and pathogenicity in GCTs were not assessed. Consequently, despite these challenges, our study represents a pivotal step toward understanding the somatic mutational landscape of pediatric germ cell tumors (GCTs) within Brazil’s admixed population.

## 5 Conclusion

Our study identified somatic variants, particularly in the *KIT* and *KRAS* genes, which are recognized as promising therapeutic targets in ongoing clinical trials for specific tumor types. These findings highlight the potential *druggability* of these genes in the context of pediatric germ cell tumors. However, further investigation in larger cohorts is essential to validate these results and fully understand their clinical implications. A deeper understanding of these molecular alterations is critical for advancing the application of cancer genomics to precision medicine, paving the way for more targeted and effective treatments in the future.

## Data Availability

The datasets presented in this study can be found in online repositories. The names of the repository/repositories and accession number(s) can be found below: https://ega-archive.org/, EGAD50000000877.

## Supplementary material

Supplementary Table 1 Individual characteristics of pediatric patients with germ cell tumors. FIGO, Classification of The International Federation of Gynecology and Obstetrics; COG, Classification of The Children’s Oncology Group; SEM, Seminoma; YST, Yolk Sac Tumor; DYS, Dysgerminoma; MT, Mature Teratoma; EC, Embryonal Carcinoma; CC, Choriocarcinoma; SC, Sexual Cord; Unc, Unclassifiable.
