# Supplementary material for: Genomic landscape of pediatric germ cell tumors reveals oncogenic mutations and copy number alterations
Source: Front Oncol. 2026 Feb 16;16:1689022. doi: 10.3389/fonc.2026.1689022 (PMC12950581; doi:10.3389/fonc.2026.1689022)
Supplement: Supplementary Table 1 — Individual characteristics of pediatric patients with germ cell tumors. FIGO, Classification of The International Federation of Gynecology and Obstetrics; COG, Classification of The Children’s Oncology Group; SEM, Seminoma; YST, Yolk Sac Tumor; DYS, Dysgerminoma; MT, Mature Teratoma; EC, Embryonal Carcinoma; CC, Choriocarcinoma; SC, Sexual Cord; Unc, Unclassifiable. [file Table1.docx]

**Supplementary Table 1.** Individual characteristics of pediatric patients with germ cell tumors.

| **Patient** | **Age (years)** | **Primary tumor** | **Histology** | **Staging** | **Metastasis** | **Relapse** | **Sample** | **Depth** |
| --- | --- | --- | --- | --- | --- | --- | --- | --- |
| **TCG_03** | 2 | Testis | YST | COG IV | Lung | No | Tumor | 446 |
|  |  |  |  | High Risk |  |  | Normal | 135 |
| **TCG_04** | 2 | Testis | YST | COG I | No | No | Tumor | 451 |
|  |  |  |  | Low Risk |  |  | Normal | 146 |
| **TCG_07+** | 9 | Mediastinum | MT | COG I | No | No | Tumor | 437 |
|  |  |  |  | Low Risk |  |  | Normal | 139 |
| **TCG_08** | 15 | Ovary | EC | FIGO I | No | Yes (ovary) | Tumor | 443 |
|  |  |  |  | Low Risk |  |  | Normal | 134 |
| **TCG_09** | 6 | Ovary | MIXED | FIGO II | No | No | Tumor | 450 |
|  |  |  | (60% CC; 40% DYS) | Intermediate Risk |  |  | Normal | 138 |
| **TCG_10** | 13 | Ovary | MIXED | FIGO III | No | No | Tumor | 452 |
|  |  |  | (50% EC; 45% YST; 5% CC) | High Risk |  |  | Normal | 147 |
| **TCG_11** | 16 | Testis | MIXED | COG IV | Lung, liver | No | Tumor | 436 |
|  |  |  | (70% EC; 25% YST; 5% MT) | High Risk |  |  | Normal | 136 |
| **TCG_12** | 9 | Ovary | MT | FIGO I | No | No | Tumor | 445 |
|  |  |  |  | Low Risk |  |  | Normal | 148 |
| **TCG_17** | 13 | Ovary | DYS | FIGO I | No | Yes (lymph node) | Tumor | 435 |
|  |  |  |  | Low Risk |  |  | Normal | 149 |
| **TCG_18** | 15 | Ovary | MIXED | FIGO IV | Lung | No | Tumor | 447 |
|  |  |  | (90% YST; 10% DYS) | High Risk |  |  | Normal | 152 |
| **TCG_19** | 13 | Ovary | MIXED | FIGO III | No | No | Tumor | 440 |
|  |  |  | (>90% YST; <1% MT) | High Risk |  |  | Normal | 137 |
| **TCG_22** | 16 | Testis | MIXED | COG IV | Lung, lymph node | Yes | Tumor | 453 |
|  |  |  | (99% MT; <1% YST; <1% EC) | High Risk |  |  | Normal | 140 |
| **TCG_23** | 3 | Ovary | MIXED | FIGO I | No | No | Tumor | 449 |
|  |  |  | (90% SC; 10% Unc) | Low Risk |  |  | Normal | 152 |
| **TCG_25** | 15 | Ovary | MT | FIGO I | No | No | Tumor | 438 |
|  |  |  |  | Low Risk |  |  | Normal | 142 |
| **TCG_29** | 17 | Testis | MIXED | COG I | No | No | Tumor | 448 |
|  |  |  | (50% SEM; 30% EC; 19% MT; 1% YST) | Low Risk |  |  | Normal | 144 |
| **TCG_30** | 18 | Ovary | YST | FIGO I | No | Yes | Tumor | 433 |
|  |  |  |  | Low Risk |  |  | Normal | 145 |

FIGO: Classification of The International Federation of Gynecology and Obstetrics. COG: Classification of The Children's Oncology Group. SEM: Seminoma. YST: Yolk Sac Tumor. DYS: Dysgerminoma. MT: Mature Teratoma. EC: Embryonal Carcinoma. CC: Choriocarcinoma. SC: Sexual Cord. Unc: Unclassifiable.
